# Sickle Cell Anaemia and Malaria

**DOI:** 10.4084/MJHID.2012.065

**Published:** 2012-10-03

**Authors:** Lucio Luzzatto

**Affiliations:** Honorary Professor of Haematology, University of Florence, Scientific Director, Istituto Toscano Tumori. Firenze. Italy

## Abstract

Sickle cell anaemia is a major chapter within haemolytic anaemias; at the same time, its epidemiology is a remarkable signature of the past and present world distribution of *Plasmodium falciparum* malaria. In this brief review, in keeping with the theme of this journal, we focus on the close and complex relationship betweeen this blood disease and this infectious disease. On one hand, heterozygotes for the sickle gene (AS) are relatively protected against the danger of dying of malaria, as now firmly established through a number of clinical field studies from different parts of Africa. In addition, experimental work is consistent with a plausibile mechanism: namely, that in AS heterozygotes *P falciparum*-infected red cells sickle preferentially and are then removed by macrophages. On the other hand, patients who are homozygous for the sickle gene and therefore suffer from sickle cell anaemia (SCA) are highly susceptible to the lethal effects of malaria. The simplest explanation of this fact is that malaria makes the anaemia of SCA more severe; in addition, in SCA there is often hyposplenism, which reduces clearance of parasites. From the point of view of public health it is important that in malaria-endemic countries patients with SCA, and particularly children, be protected from malaria by appropriate prophylaxis.

The history of sickle cell anaemia (SCA) lists several gold medals. First, it was for SCA that the term molecular disease was coined over half a century ago^1^, and this led to the notion of haemoglobinopathies. Second, when the structural abnormality of haemoglobin (Hb) S was pinpointed^2^, this was the first time that a single amino acid replacement in a protein was shown to cause a serious disease. Third, once the three-dimensional structure of Hb was solved^3^ it became clear why Hb S had the unique characteristic of being normal when oxygenated, but abnormal when deoxygenated.^4^,^5^ Fourth, once the globin genes were cloned, the sickle mutation was found to be in linkage disequilibrium with a polymorphic DNA site,^6^ then called a restriction fragment length polymorphism (RFLP), now called a SNP: this was the seminal principle on which all of today’s genome wide association studies (GWAS) are based.

Thus, the entire field of human molecular genetics is greatly indebted to SCA; at the same time, as far as haematology is concerned, SCA is a major chapter within haemolytic anaemias.

Here we intend to discuss briefly one aspect of this condition that is eminently germane to the very name of this journal: we focus on where SCA, a blood disease, meets malaria, an infectious disease. The relationship is complex. Here we will try to briefly pinpoint what we know and what we don’t yet know about this two-way relationship: malaria has influenced greatly the epidemiology of SCA, and SCA affects the clinical course of malaria.

## The ‘Malaria Hypothesis’

That different persons may differ in how they respond to an infectious disease has been probably perceived for a long time. However, the first to formulate this notion in terms of Darwinian selection was J B S Haldane, who speculated that, depending on their genetic makeup, people would have a different risk of dying when they are confronted by a parasitic organism: so much so, that even if a gene offering protection against that parasite were otherwise harmful, its frequency would increase when a population was exposed to the parasite.^7^ Haldane himself later hypothesized^8^ that one important example could be thalassemia in the face of malaria, for several reasons. First, one type of malaria, that caused by *Plasmodium falciparum*, is highly lethal. Second, it is estimated to have been around in many parts of the world for several thousands of years, *i.e.* for several hundreds of generations: thus, malaria as an agent of natural selection seemed a better candidate than an infectious disease causing occasional epidemics even if associated with high mortality (such as plague or influenza). Third, deaths from malaria take place mostly in children, *i.e.* before reproduction, a critical criterion for effective selection. Last but not least, *Plasmodia* take on different forms in the course of their life cycle, but what causes disease are the intra-erythrocytic parasites: therefore in principle it is not surprising that if red cells are in any way abnormal (as they, are, for instance, in thalassemia), this may affect the chance of success of the parasite.

## Balanced Polymorphism

Many fundamental experiments in genetics have been carried out in micro-organisms, and biological selection is a good example. Growing bacteria in a culture medium containing streptomycin is a very simple and certain way to select for the few bacteria, within the culture, that already had a gene – we can call it *str^r^* – that makes them resistant to this antibiotic. If we now isolate one of the resistant bacteria we can grow up a new culture in which the entire population will be streptomycin resistant. It happens that the streptomycin-resistant bacteria do not grow quite as fast as the streptomycin-sensitive ones:^9^ thus, in the presence of streptomycin the *str^r^* gene is a great advantage; in the absence of streptomycin it is a disadvantage. Since bacteria are mostly haploid (*i.e.* they have only one copy of each gene), each one of them either has the *str^r^* gene or it doesn’t: there is nothing in between.

Since we humans, like most animals, are diploid, we have in this respect more options. SCA is a disease of homozygotes (SS) – this is why we call the disease recessive – whereas heterozygotes (AS) are normal for most intents and purposes. The first test of Haldane’s hypothesis was carried out by A C Allison,^10^ when he showed not only that the S gene was frequent in areas of high malaria transmission, but also that AS heterozygotes seemed to have less malaria. By the laws of population genetics it is to be expected that wherever the S gene is common there will be many patients suffering from SCA, a severe burden in the population; however, in the same population a much larger number of heterozygotes (see Table 1) will have the advantage of being, in first approximation, ‘malaria-resistant’. The disadvantage of homozygotes coexisting with the advantage of heterozygotes –therefore called a *balanced polymorphism –* had been already well characterized in *Drosophila*^11^ and in other model systems: with the S gene it became clear that balanced polymorphism was a reality also in the human species.

## How the S Gene Affects Malaria

Allison’s seminal work has been abundantly confirmed by numerous studies on much larger population samples validated by rigorous statistical analysis;^12^ and they have indicated that for AS heterozygotes the phrase ‘malaria-resistant’ ought to be regarded as shorthand for ‘relatively protected from dying of malaria’. In essence, the following points have emerged. (a) AS heterozygotes do get malaria. (b) AS heterozygotes with malaria tend to have lower numbers of parasitized red cells in their blood. (c) AS heterozygotes have a decreased incidence of the two forms of severe malaria recognized as immediately life-threatening: namely, cerebral malaria and malaria with severe anaemia. (d) Very rarely do AS heterozygotes die of malaria, even in the rare cases when they do develop cerebral malaria.^13^ It stands to reason that (d) is a consequence of (c), and (c) is at least to some extent the result of (b).

**Figure 1:**
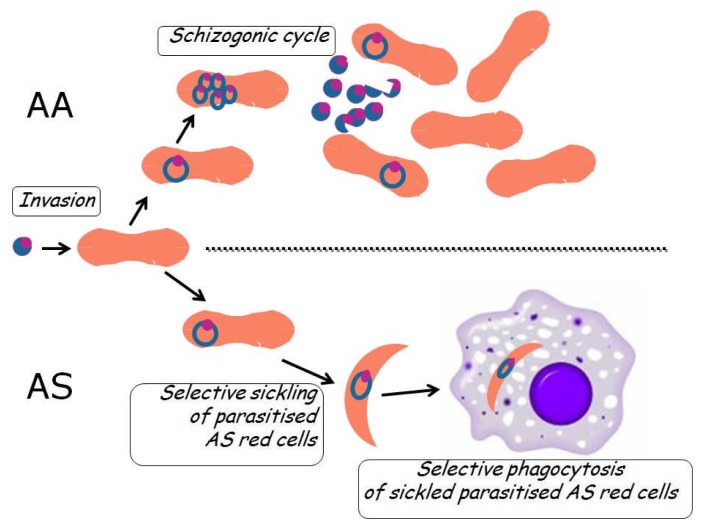
*Cartoon illustration of how AS heterozygotes are relatively protected from severe* P falciparum *malaria.* The upper part of the cartoon is a schematic diagram of what happens in red cells in a normal (Hb AA) person with malaria: after invasion of a red cell by a *merozoite*, this becomes a *ring form*, and this starts multiplying (*schizogony*) ; when a *schizont* is mature the infected red cell essentially bursts and releases new merozoites, each one of which can invade a new red cell. The lower part of the cartoon is a schematic diagram of what happens in red cells in an AS heterozygote with malaria: the red cell, which appears normal at the time of invasion, once infected undergoes sickling (probably as a result of deoxygenation and lowering pH caused by the parasite), and thus it falls easy prey to macrophages in the spleen, in other organs and even in the peripheral blood^42^. Phagocytosis of a parasitized red cells clearly interrupts the schizogonic cycle and thus the parasitaemia can be kept under control.

These data from clinical epidemiology^14^ are consistent with increased fitness of AS heterozygotes in an environment with malaria (there would be no advantage in an environment without malaria); at the same time, they tell us clearly that Hb S is not an absolute impediment to the malaria parasite. Therefore, the mechanism for the increased fitness of AS heterozygotes is not failure of invading red cells (see Table 2): rather, it must be based in something that takes place subsequently. Beet^15^ first suggested that the phenomenon of sickling may be responsible; and subsequently it was shown by quantitative *in vitro* studies that the rate of sickling of AS red cells that had been parasitized *in vivo* was significantly higher than that of non-parasitized red cells within the very same blood sample.^16^ It seemed reasonable to surmise and it was shown subsequently (see Figure 1) that once the parasite has triggered sickling the sickled cells would be removed by macrophages^17^.

This mechanism is consistent with *in vitro* culture studies that have shown normal growth of *P falciparum* in AS red cells and even in SS red cells,^17^,^18^ clearly indicating that it is not Hb S *per se* that hinders parasite development: it must be something downstream of the parasite cycle, such as phagocytosis of sickled cells.

In fact, although it is often stated that the mechanism of protection against malaria of AS heterozygotes is not clear, over the past 40 years there has not been any evidence contrary to the sickling-phagocytosis model; and increased phagocytosis of AS parasitized red cells has been confirmed.^19^ The clinically relevant consequence of this process is to keep parasitemia relatively low in AS heterozygotes, and this has been also abundantly confirmed in many studies.^14^,^20^–^23^ Of course there may be other protective mechanisms at work: for instance, it has been found that AS parasitized red cells have impaired adherence to endothelial cells, which could decrease the risk of cerebral malaria.

The impaired cytoadherence seems to result from altered display on the red cell surface of the *P falciparum* erythrocyte membrane protein 1 (PfEMP-1).^24^ Very recently, through elegant cryoelectron tomography microscopic techniques it has been shown that PfEMP-1 display depends on remodeling by the parasite of the red cells cytoskeleton; and that this process is defective in CC and SC red cells^25^ (AS red cells have not yet been tested). Protection against malaria by the S gene has been also demonstrated in a mouse model, and attributed to accelerated breakdown of haeme by haeme oxygenase^26^ however, the pathophysiology of *P berghei* malaria in mouse is very different from that of *P falciparum* in humans, and therefore it is difficult to know whether this interesting phenomenon observed in the former is relevant to the latter.

**Figure 2:**
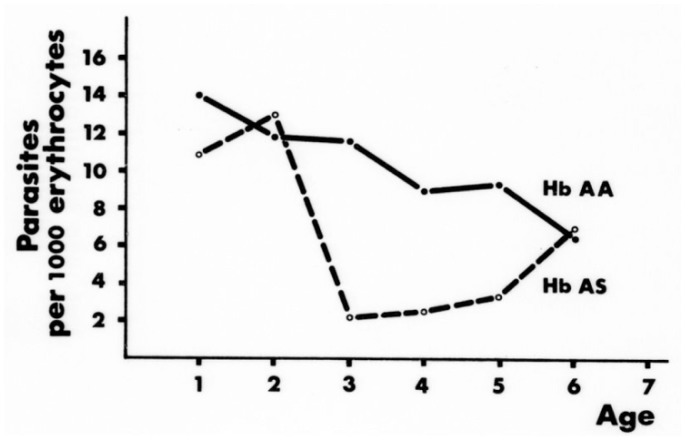
*In an area of heavy malaria (Abeokuta, SW Nigeria) the* P falciparum *parasite density is significantly reduced in AS versus AA children, specifically between the age of 3 and 5.* Protection from life-threatening levels of parasitaemia is crucially important in this age group for the survival of AS heterozygotes, because subsequently acquired immunity can protect AA subjects as well. From.^20^

Acquired immunity is a major determinant of the clinical outcome of malarial infection. Several studies have suggested that AS heterozygotes have accelerated acquisition of immunity,^27^,^28^ although the matter is still controversial.^29^ A recent study carried out in Uganda has shown that AS heterozygous children (age 1–10) are protected from (i) the establishment of blood-stage infection, (ii) the development of high densities of parasites, (iii) the progression of infection to symptomatic malaria.^30^ From an analysis of data as a function of age the authors infer that both innate and acquired mechanisms of protection come into play. This confirms the notion 20 that the main advantage of AS heterozygotes in areas with heavy malaria endemicity consists in their increased probability of surviving until acquired immunity is sufficient to protect them, as well as others, regardless of their haemoglobin type (Figure 2).

## How Malaria Affects Patients with SCA

If AS heterozygotes were protected from malaria through failure of infection, one might expect protection to be at least as effective in SS homozygotes, i.e. in patients with SCA: however, the mechanism is not failure of infection, and therefore it may not necessarily apply to homozyogotes SCA patients. They have a prototype congenital haemolytic anaemia and are susceptible to malaria, which is a prototype acquired haemolytic anaemia. Clinical experience has shown that, not surprisingly, this combination is highly dangerous.^31^ One obvious reason is that malaria will make the anemia of SCA worse, to the point of it becoming life-threatening; another reason is that malaria, like any other acute infection, can trigger in a patient with SCA a pain crisis or a sequestration crisis. Of special note is the fact that normally the spleen plays an important role in filtering and removing parasitized red cells: but patients with SCA regularly have an impaired splenic function: often to the extent of functional asplenia, and sometimes the functional asplenia evolves to anatomical atrophy of the spleen from multiple infarcts (so-called auto-splenectomy).^32^ A recent population study carried out in Kenya has shown that malaria is no more common in SCA children than in controls: however, the mortality of SCA children who had malaria was about 10 times higher than in controls.^33^ We can infer that in Africa malaria contributes substantially to the early mortality of patients with SCA, which makes it imperative that they ought to be protected by life-long antimalarial prophylaxis.

## Conclusion

In an era of evidence-based medicine it is still not uncommon that hypothetical propositions are stated as facts. By contrast, it is quite remarkable that the protective effect of the Hb S gene against malaria is still portrayed as a hypothesis when it is, in fact, one of the best documented examples in the human species of balanced polymorphism, in which the severe disease of homozygotes (SS or SCA) is balanced by the advantage of AS heterozygotes.

Malaria and sickle cell anaemia are still major challenges to infectious disease medicine and to haematology respectively, and both are also major public health problems. One might have hoped that what we have learnt about the of advantage of AS heterozygotes with respect to malaria would enable us to protect from malaria mortality other people as well. That this has not yet happened is disappointing but perhaps not surprising, because the key is sickling of red cells, and this is a unique phenomenon. It cannot be a straightforward task to mimic sickling by a pharmacological approach in subjects who do not have Hb S, and in a way that would act selectively only on parasitized red cells. We can still hope that human imagination will evolve novel approaches that can match the power of mutation and selection in biological evolution. In the meantime SCA remains a source of great suffering to patients, especially in those developing countries where the numbers are staggering (see Table 1). It is urgent that more is done in order to offer to these patients a better way of life: this ought to include optimal management of pain, often hydroxyurea and, especially in Africa,^34^ protection against the potentially fatal threat of *P falciparum* malaria. If, as doctors, we have a professional obligation towards all of our patients, for those with SCA we have an added human obligation, if we consider that they carry the genetic burden that has helped human populations to survive in malaria-endemic regions of the world.

## Figures and Tables

**Table 1:** Theoretical and real life examples in the epidemiology of the sickle cell trait and of sickle cell anaemia

	Hypothetical region/country			Nigeria
	A	B	C	
Population, millions	5	25	25	156^a^
Frequency of Hb^S^ allele	0.10	0.01	0.07	0.11 b
Number of AS heterozygotes, millions	0.9	0.495	3.255	30.5
Predicted frequency of SS patients, % c	1	.01	.49	1.21
Number of SS patients d	50,000	2,500	122,500	1,887,600

a Population of Nigeria in year 2010 according to http://www.indexmundi.com/nigeria/population.html

b Approximate average estimate from values in different parts of the country^35^

c Calculated on the basis of Hardy-Weinberg equilibrium

d Calculated from the data in the line above: figures are over-estimates in view of the considerable early mortality of SS patients

**Table 2:** Protective mechanisms against malaria deployed by polymorphic genes expressed in red cells

*Basic Mechanisms*	*Example*	*Comments*	*References*
1. Failure of invasion	*P vivax* in Duffy-negative red cells	*P vivax* not found in West Africa where almost all people are Fy−/−	36
2. Impaired intra- erythrocytic growth	*P fal* in Hb CC red cells	Haemoglobin C interferes with ability of parasite to remodel host cell cytoskeleton	25; 37; 38
3. Enhanced removal of parasitized red cells	Hb AS red cells sickle preferentially when they are *P fal* infected	*Suicidal infection*^39^: parasitised sickled cells are phagocytosed (see Fig. 2). Probably applies also to parasitized G6PD deficient red cells	16; 40; 41
